# Number of syllables in cuckoo *Cuculus canorus* calls: A test using a citizen science project

**DOI:** 10.1038/s41598-018-31329-1

**Published:** 2018-08-27

**Authors:** Yanina Benedetti, Karolina Slezak, Anders Pape Møller, Federico Morelli, Piotr Tryjanowski

**Affiliations:** 1 Faculty of Environmental Sciences, Department of Applied Geoinformatics and Spatial Planning, Czech University of Life Sciences Prague, Kamýcká 129, CZ-165 00 Prague 6, Czech Republic; 2 Institute of Zoology, Poznań University of Life Sciences, Wojska Polskiego 71C, 60-625 Poznań, Poland; 3 Ecologie Systématique Evolution, Université Paris-Sud, CNRS, AgroParisTech, Université Paris-Saclay, F-91405 Orsay Cedex, France

## Abstract

Recent studies revealed that the call of the common cuckoo *Cuculus canorus* has more inter-individual than intra-individual variation and that the number of syllables depends on environmental conditions, but also the presence of male and female conspecifics. However, still very little is known about how song varies at a global scale, especially considering the wide distribution of this species across most of Europe and Asia. Xeno-canto.org is a vocalization repository for birdsong. We used xeno-canto.org as a data source for investigating the variables that affect the number of syllables in cuckoo calls at a large spatial scale. At a very broad geographical scale, the number of syllables in cuckoo calls predicted bird species richness. Additionally, female calls were associated with shorter males calls, and there was a positive correlation between the interaction between female calls and the number of host races parasitized by the cuckoo. These findings confirm that intraspecific and interspecific interactions significantly affect the number of syllables in cuckoo calls, and both environmental variables and biotic interactions should be considered in future studies of vocalizations in cuckoos. Last but not least, we demonstrated that a citizen science project is a useful source for ecological studies at large spatial scales.

## Introduction

The common cuckoo *Cuculus canorus* (henceforth cuckoo) has fascinated humans since millennia^1^. It is an obligate brood parasite occurring throughout Europe and Asia, from England to Japan^2–4^ with a very large population size^5^. Still, its population trend is decreasing^6^. However, the species does not reach the threshold for it to be considered Vulnerable in the Red List categories^5^. The cuckoo has parasitized more than 200 different bird species^3^. The occurrence of cuckoos reflects overall species richness of their hosts. For this reason, the cuckoo has recently been suggested to reliably indicate biodiversity^7–9^. However, most importantly, the call of the cuckoo is recognized from its voice by the general public^10^. As a consequence, the cuckoo’s name evokes its typical call in numerous languages since thousands of years^11,12^.

Several studies have determined the relevance of auditory and visual signals in intraspecific and interspecific interactions in birds. Generally, at the intraspecific level, birds use calls and plumage to compete for a territory or acquire a mate^13–15^. Birds also use auditory and visual signals during predator–prey interactions^16^. The male cuckoo is much more vocal than the female, and it produces a very recognizable “cuck-ooo” call during the breeding season, consisting of two notes that are repeated many times^17^. Recent studies revealed that cuckoo calls vary more among than within individuals^18,19^. Furthermore, Møller *et al*. (2016) highlighted that the number of syllables in male cuckoo calls differs depending on environmental characteristics such as soil type, habitat, level of environmental radioactivity and the presence or the absence of male or female conspecifics.

Collection of data at continental and global scales produces hundreds of millions of observations of thousands of species every year^20^. From websites and social media to smartphones, apps, low-cost sensors and search engines data capture become easier and faster^21,22^. The recent availability of ‘big data’ associated with predictive analytics have profound implications for biological recording activities. An example of globally disseminated resources is www.xeno-canto.org (Xeno-canto, XC), a non-profit website set up to share recordings of bird sounds worldwide^23,24^. Its main goal is to improve knowledge of bird sounds by increasing accessibility and diffusion of bird sound recordings from across the globe^25^. Thereby, Xeno-canto data can help improve our knowledge of the complexity of cuckoo calls, and more specifically, the factors that cause such variation on a broader global scale.

The objectives of this study were to use a dedicated media and citizen science sources to explore at a wider spatial scale (1) the factors that influence the number of syllables in cuckoo calls, (2) whether the number of syllables of cuckoo calls reflects bird species richness, and (3) if cuckoo call activity is determined by environmental factors. Finally, we discuss how dedicated media sources can provide useful scientific data and thereby contribute to ecology and conservation.

## Results

**Figure 1:**
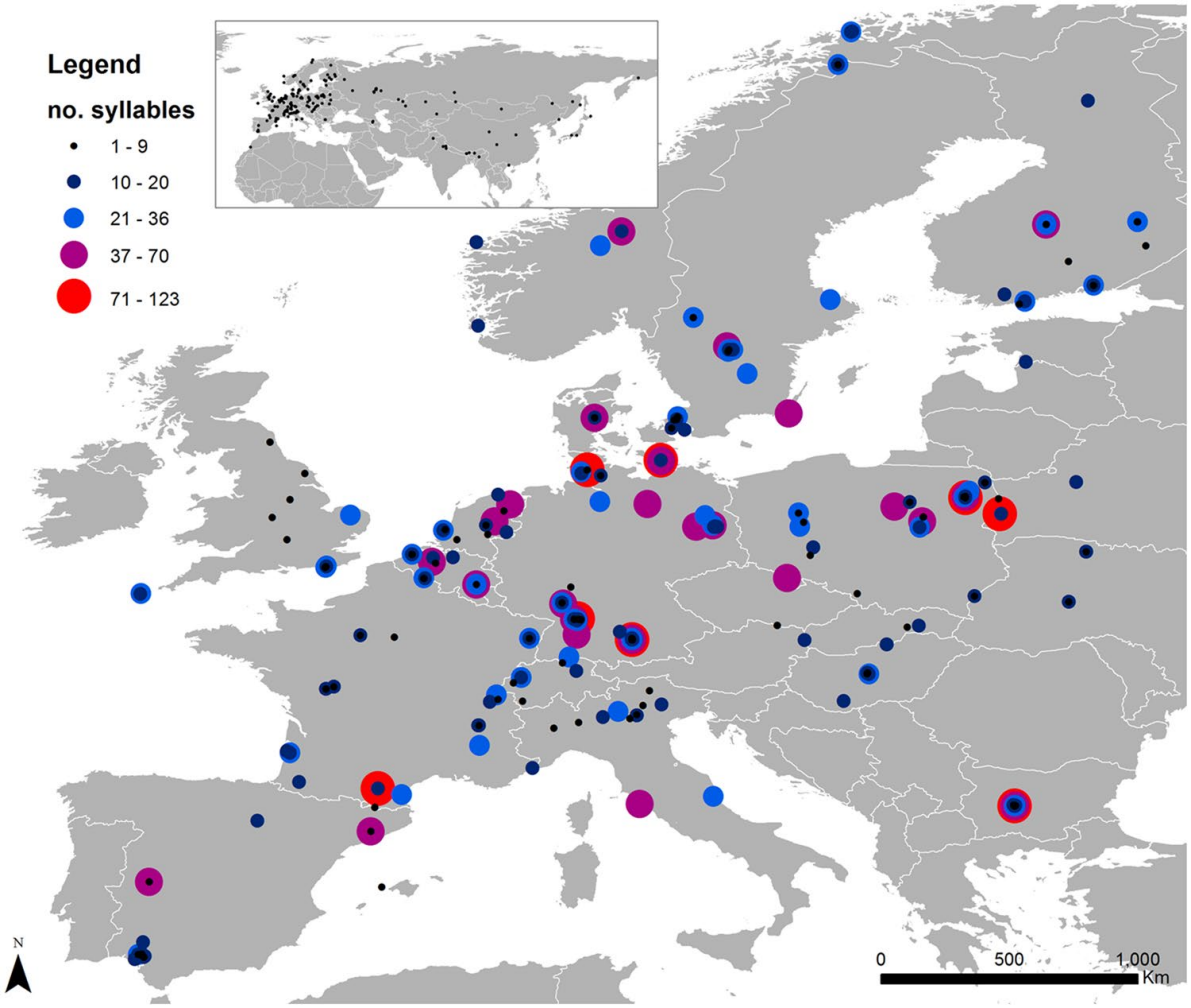
Geographical distribution of the number of syllables in male cuckoo calls obtained from the Xeno-canto website. The map was produced with GIS soſtware (ArcGIS 10.1)^58^ with free geographic background data. The geographical distribution of cuckoo *Cuculus canorus* calls was mapped using freely available data from http://www.xeno-canto.org.

From the Xeno-canto database source, we identified and analysed 484 call recordings of the common cuckoo from 29 different countries in Europe, Asia and Africa (Fig. 1). Most records were from Germany (21.0%), Bulgaria (12.4%) and France (9.1%). Call recordings were registered in 22 different years during 1982–2016. More than 60% of call recordings were registered from 2014 to 2016.

### Cuckoo call activity patterns

Cuckoo calls were recorded practically throughout the day (ESM, Fig. 1). The time of the day with more recordings was between 04:00 to 08:00 (27.5% of recordings). Conversely, from 12:00 to 16:00 cuckoo calls reached a minimum at 8.9%. Cuckoo calls were also registered during the night between 20:00 and 04:00 (12.8%) (ESM, Fig. 1). The number of syllables of cuckoo calls was not significantly associated with the date, time of day and duration of records.

### Number of syllables of cuckoo calls and bird species richness

Each call recorded was composed on average of 16.99 syllables (max: 123, min: 1, SD: 16.7), and the duration of these records was on average 150.1 seconds (max: 1202.7, min: 3.2, SD: 236.23). A total of 99 bird species were heard in the background when cuckoo calls were recorded of which 49.5% were cuckoo hosts (ESM, Table 1). The number of syllables in cuckoo calls was positively correlated with bird species richness (Table 1).

**Table 1:** Results of fixed-effect parameters in GLMM, accounting for variation in variables in relation to female recording, date, hour, duration of recording, number of host races, population cuckoo density, species richness and latitude.

Variable	ES	SE	*t*	*P*
Intercept	2.444	1.028	2.378	<0.05
**Female recording**	**−2.968**	**0.817**	**−3.633**	**<0.001**
Date	−3.8e-4	0.004	0.097	>0.05
Hour of day	−0.454	0.392	−1.158	>0.05
Duration of record	−3.1e-4	−3.2e-4	0.982	>0.05
Number of host races	−2.1e-2	0.029	−0.709	>0.05
Population density	0.245	0.373	0.658	>0.05
**Bird species richness**	**0.081**	**0.039**	**2.075**	**<0.05**
Female recording*number of host races	**0.234**	**0.094**	**2.490**	**<0.05**
Latitude	2.3e-3	0.015	0.150	>0.05

The interaction between years and countries was added as a random factor in the models. Significant variables are shown in bold. Abbreviations: ES, Estimate; SE, standard error.

### Intraspecific influence on number of syllables in cuckoo calls

**Figure 2:**
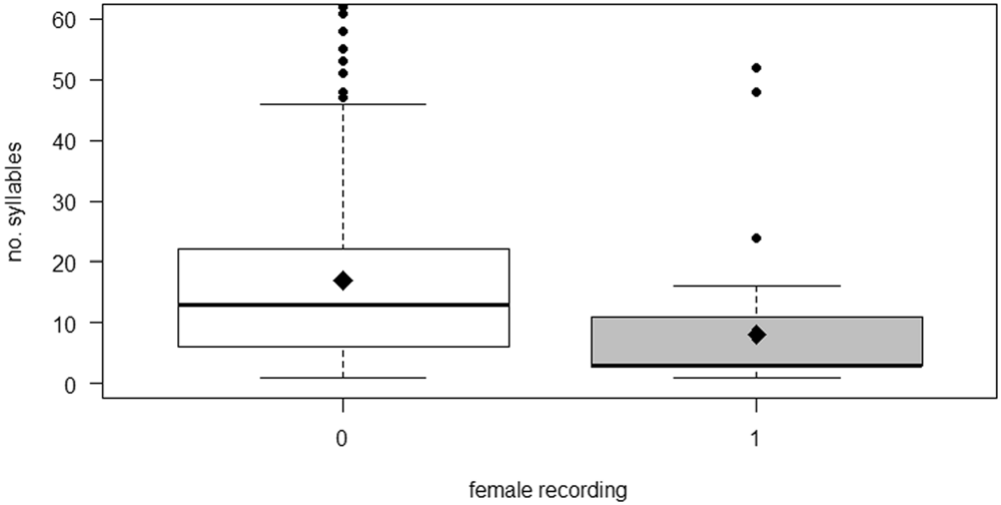
Number of syllables in male cuckoo calls in relation to the presence of a female call. The box plots show medians, quartiles, 5- and 95-percentiles and extreme values.

Female cuckoos produced calls in 7.85% of cases when male calls were recorded, while the number of syllables was negatively correlated with the presence of female cuckoo calls (Fig. 2, Table 1). The number of syllables was positively correlated with the interaction between female calls and the number of host races, but it was not significantly correlated with the other variables (number of host races and population density) (Table 1).

## Discussion

The opportunity for access to a huge amount of data from social media and citizen science projects is changing the way in which scientific studies and analyses of biological records are performed. Such studies allow for analyses at broad spatial and temporal scales, as well as promising to connect the efforts of experts and non-experts in science^20^. We explored the characteristics of the calls of male cuckoos based on long-term recordings and calls from across a wide geographical range from the online website Xeno-canto.org.

Although the calls of the common cuckoo from the Xeno-canto database derive across Europe, Asia and Africa, most recordings were uploaded from Germany, Bulgaria and France. A similar heterogeneous source of data was observed in a project studying the song dialects of the yellowhammer *Emberiza citronella*^26^. These findings suggest that big data sources from citizen science projects can be influenced by particular amateur preferences (for species and/or habitats). Such preferences can potentially cause a bias if statistical analyses do not explicitly pay attention in order to minimize such bias.

The characteristics of the cuckoo call have for a long time played an important role in popular folklore. Thus, its common name is very similar among numerous languages, often associated with the particular vocalization of this bird^11^. The number of syllables in cuckoo calls is correlated with environmental variables and intraspecific interactions^27,28^. The log-normal distribution of the number of syllables reported here matches that of previous studies. This indicates that most recordings have calls with few syllables. However, the range in the number of syllables per call reported here was much more variable than in long-term studies in Ukraine and Denmark^27,28^.

It is often assumed that the timing of bird calls is related to weather and the function of calls. Therefore, passerines with calls having a function in the context of sexual selection sing mainly in early morning and at dusk^14^. The calls of the common cuckoo have not only a function in sexual selection, but may also affect potential hosts^29,30^. We hypothesize that the timing of calls fits the activity of hosts. The Xeno-canto database consisted of cuckoo calls recorded throughout the day. However, most calls were from early morning to midday, while cuckoo calls reached a minimum in the afternoon similar to the diurnal pattern of singing in other bird species^14^. This global pattern is also similar to the diurnal pattern of cuckoo calls in the Chernobyl area^27^. Interestingly, cuckoo calls were also recorded during the night and early morning, as reported in the literature^31–33^.

Cuckoo males produced calls with more syllables when a female cuckoo was present, but also when another male was present^27^. In contrast, the number of syllables in male cuckoo calls was strongly negatively correlated with the number of syllables in recordings of female cuckoos. However, it is possible that the recordings mainly focused on female calls, but that the calls of males from the vicinity were also heard. However, we cannot exclude the possibility that females are present without producing any calls.

Host-race formation or speciation takes place when a new host is exploited by a parasite, and the phenotype of cuckoo eggs eventually is adapted to those of the host^34,35^. Therefore, a brood parasite as the common cuckoo is divided into host races, each characterized by egg mimicry of different host species^35–38^. In our study, the number of syllables in cuckoo calls was positively correlated with the interaction between female recordings and the number of host races. This fact suggests that the number of syllables in cuckoo calls may reflects the already known relationship between host races and calls of female and male cuckoos, where female cuckoos show strong host preferences, while individual males mate with females that lay in the nests of different hosts^34^.

Previous studies have already shown that the presence of the cuckoo and its population density are reliable predictors of overall bird diversity, especially of cuckoo hosts^7,8^. Here we have shown that the number of syllables in cuckoo calls is positively correlated with bird species richness. Moreover, Møller *et al*. (2017) suggested that cuckoo calls are reliable signals of habitat quality expressed as the number of breeding bird species. Here we extended this pattern to a broad geographical scale, across the entire geographical range of the cuckoo.

In conclusion, a citizen science project using data from Xeno-canto.org allowed collection of a large number of cuckoo calls from Europe, Asia and Africa. When we analyzed Xeno-canto data we found that cuckoo call characteristics were related to the number of syllables of cuckoo calls and species richness (i.e. a positive correlation with bird species richness). There was also an intra-specific positive relationship between the interaction between female calls and the number of hosts races. Previous ecological studies used big data from citizen science and social media projects^39,40^, and these studies suggest that social media are changing the approach used in ecological and conservation research.

## Methods

### Data collection

We used Xeno-canto (http://www.xeno-canto.org/) as a data source for common cuckoo call recordings. Even if Xeno-canto mainly focuses on male cuckoos, females are occasionally recorded. Each recording was analysed and the following data were extracted from the dataset: sex (male; female if any; however we have to emphasize that normally recordings were dedicated to males, and that only sometimes were female calls recorded, confirming that the female was present close to the male); number of syllables; time of day (min:sec); date (year-month-day); geographical location (country, GIS coordinates: latitude, longitude); bird species in background; observer/recorder name and catalogue number.

### Definition of variables

#### Number of syllables

**Figure 3:**
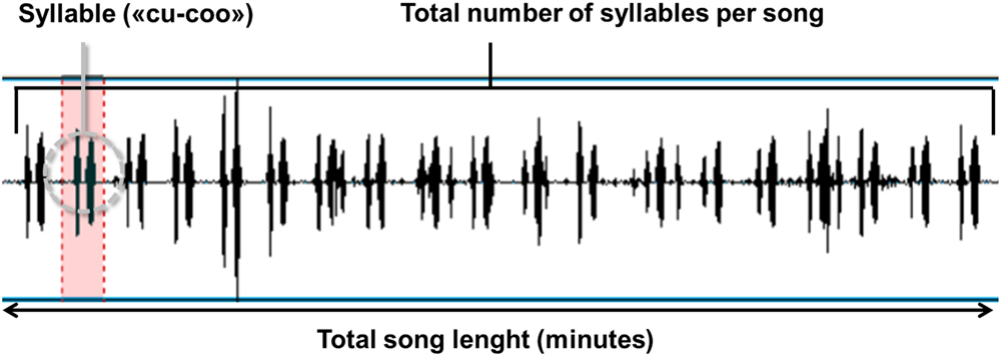
An example of a sonogram with the count of the number of syllables in a male cuckoo song. The sonogram was prepared by Møller^28^.

We explored the number of syllables that a cuckoo male produces in a single call for each record^27,28^ (see Fig. 3). The number of syllables defined as each syllable being a single “*cu-coo*” was counted by waiting for a call to be finished in order to avoid inclusion of overlapping calls. A call was considered to be finished when at least a period of time similar to that of a syllable had passed without a new syllable being produced. We also counted the number of syllables in the subsequent two calls.

#### Bird species richness

Bird species richness was the number of bird species singing in the background in each cuckoo call recording.

#### Cuckoo host species

The list of host species of the cuckoo was recorded from the following sources^3,4,36,37,41^

#### Population density of cuckoo

We estimated the population sizes of cuckoos for different countries as reported by Burfield and van Bommel^42^. Population sizes were reported as a range, and we used the mean for the analyses.

#### Cuckoo host races and their distribution

The common cuckoo has several host races, each classified by egg mimicry of different host species^34,43,44^. Moksnes and Røskaft^4^ categorized all cuckoo eggs produced by 17 different host races based on color and spottiness of the egg (location, color, density and size of spots). Besides, Møller *et al*.^38^ added to the compilation of host races by Moksnes and Røskaft five additional host races recently described^38,45–47^. Thus, we classified the distribution of all 22 host races of the cuckoo in the 28 European countries.

### Statistical analysis

We tested for spatial auto-correlation (SAC) of the data by using Mantel tests^48^, based on Monte Carlo permutations (999 randomizations) to test for statistical significance^49^. In this study, the Mantel statistic r_M_ evaluates the similarity between the number of syllables among sample sites and the geographical distance among these sites^50^. Study sites were treated as statistically independent observations because the spatial autocorrelation in the data was not significant (r_M_ = −0.033, p = 0.843, n randomizations = 999).

We performed a principal components analysis (PCA) in order to avoid redundancy among variables, as a potential cause of multicollinearity, thereby removing strongly correlated predictors^51^. From PCA analysis we selected the following eight descriptors: female recording, date, hour of day, duration of record, number of host races, cuckoo population density, species richness and latitude, adequate to describe the effects of the number of syllables of cuckoo calls.

We used Generalized Linear Mixed Models (GLMM), using the package ‘lme4’^52,53^ to explore variation in cuckoo calls in relation to environmental variables and inter- and intraspecific characteristics. The number of syllables was log_10_-transformed and models were fitted supposing a log-normal distribution after exploration of the distribution of this variable, as suggested by Box and Cox^54^ using the package ‘MASS’^55^. The number of syllables in cuckoo calls was used as the response variable, while female call, date, hour of day, number of host races, cuckoo population density, bird species richness and latitude and longitude were used as predictors. The interaction between year and country were added as a random factor in the statistical model. The ‘best’ model explaining greater variation in data was assessed using Akaike’s Information Criterion (AIC)^56^. Confidence intervals for the significant variables were calculated by the Wald method, using the package ‘MASS’^55^. All statistical tests were performed with R software^57^.

## Electronic supplementary material

Supplementary material

## Data Availability

The datasets generated during and/or analysed during the current study are available from the corresponding author on reasonable request.
