# Supplementary material for: Number of syllables in cuckoo Cuculus canorus calls: A test using a citizen science project
Source: Sci Rep. 2018 Aug 27;8:12872. doi: 10.1038/s41598-018-31329-1 (PMC6110805; doi:10.1038/s41598-018-31329-1)
Supplement: Supplementary file 1 — Supplementary material [file 41598_2018_31329_MOESM1_ESM.pdf]

Number of syllables in cuckoo *Cuculus canorus* calls: A test using a citizen science project

Yanina Benedetti[1,*], Karolina Slezak[2], Anders Pape Møller[3], Federico Morelli[1], Piotr Tryjanowski[2]

[1] *Faculty of Environmental Sciences, Department of Applied Geoinformatics and Spatial Planning, Czech University of Life Sciences Prague, Kamýcká 129, CZ-165 00 Prague 6, Czech Republic*

[2]*Institute of Zoology, Poznań University of Life Sciences, Wojska Polskiego 71 C, 60-625 Poznań, Poland*

[3] *Ecologie Systématique Evolution, Université Paris-Sud, CNRS, AgroParisTech, Université Paris-Saclay, F-91405 Orsay Cedex, France*

Word count: 3362

*Corresponding author: ybenedetti73@gmail.com; +420774685349

Running headline: Citizen science and number of syllables in cuckoo calls

Legends to table and figure

Table 1. List of bird species identified in the background on cuckoo call recordings and indication with an “X” if these species are common cuckoo *Cuculus canorus* hosts. The latter information was obtained from Davies, 2000; Erritzøe et al., 2012; Moksnes and Røskaft, 1995; Payne, 2005; Yang et al., 2012

Fig. 1. Distribution of cuckoo call recordings during different intervals of the day.

Table 1

Identified bird species on call records	Hosts species of *Cuculus canorus*
Acrocephalus arundinaceus	x
Acrocephalus orientalis	
Acrocephalus palustris	x
Acrocephalus schoenobaenus	x
Acrocephalus scirpaceus	x
Actitis hypoleucos	
Alauda arvensis	x
Anser anser	
Anthus trivialis	x
Ardea cinerea	
Botaurus stellaris	
Caprimulgus europaeus	
Carduelis carduelis	x
Carduelis chloris	x
Carduelis serinus	
Carpodacus erythrinus	
Certhia brachydactyla	x
Chloris chloris	x
Chroicocephalus ridibundus	
Coccothraustes coccothraustes	x
Columba palumbus	
Corvus corax	
Corvus cornix	
Corvus corone	
Coturnix coturnix	
Crex crex	
Cuculus canorus	
Cuculus orientalis	
Cyanistes caeruleus	
Cyanoptila cyanomelana	

Dendrocopos major	
Dryocopus martius	
Emberiza calandra	x
Emberiza citrinella	x
Emberiza hortulana	x
Emberiza schoeniclus	
Emberiza spodocephala	x
Erithacus rubecula	x
Ficedula albicilla	
Ficedula hypoleuca	x
Ficedula parva	x
Fringilla coelebs	x
Fringilla montifringilla	x
Fulica atra	
Gallinago gallinago	
Hirundo rustica	x
Lanius collurio	x
Limosa limosa	
Linaria cannabina	
Locustella fluviatilis	
Locustella luscinioides	x
Locustella naevia	x
Lophophanes cristatus	
Loxia curvirostra	
Luscinia luscinia	
Luscinia megarhynchos	x
Luscinia svecica	x
Lyrurus tetrix	
Monticola saxatilis	x
Motacilla alba	x
Muscicapa striata	x

Nucifraga caryocatactes	
Oriolus oriolus	x
Parus major	x
Passer domesticus	x
Passer montanus	x
Periparus ater	
Periparus ater melanolophus	
Phasianus colchicus	
Phoenicurus phoenicurus	x
Phyllocopus borealis	
Phylloscopus bonelli	
Phylloscopus collybita	x
Phylloscopus humei	
Phylloscopus trochilus	x
Picus viridis	
Podiceps cristatus	
Poecile montanus	
Poecile palustris	
Pucrasia macrolopha	
Regulus regulus	
Remiz pendulinus	x
Saxicola rubicola	x
Spinus spinus	
Sterna hirundo	
Streptopelia orientalis	
Streptopelia turtur	
Sturnus vulgaris	x
Sylvia atricapilla	x
Sylvia borin	x
Sylvia communis	x
Sylvia nisoria	x

Tringa nebularia	
Troglodytes troglodytes	x
Turdus iliacus	x
Turdus merula	x
Turdus philomelos	x
Turdus pilaris	x
Turdus viscivorus	x

Figure 1

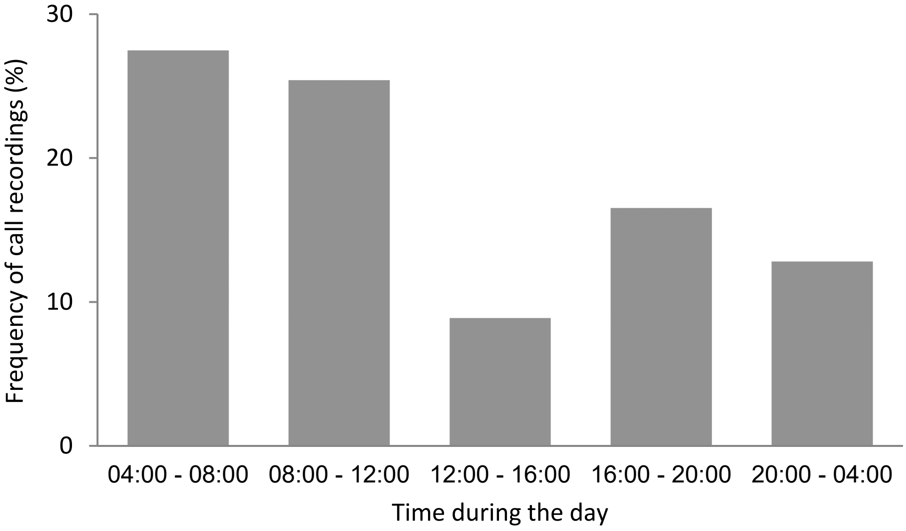

30
20
10
0
Frequency of call recordings (%)
04:00 - 08:00
08:00 - 12:00
12:00 - 16:00
16:00 - 20:00
20:00 - 04:00
Time during the day

References

Davies, N.B., 2000. Cuckoos, cowbirds and other cheats. T & AD Poyser Ltd, London, UK, London.

Erritzøe, J., Mann, C.F., Brammer, F.P., Fuller, R.A., 2012. Cuckoos of the World. Christopher Helm, London, UK, London, UK.

Moksnes, A., Røskaft, E., 1995. Egg morphs and host preference in the common cuckoo (Cuculus canorus): An analysis of cuckoo and host eggs from European museum collections. J. Zool. 236, 625–648.

Payne, R.B., 2005. The Cuckoos. Oxford University Press, New York.

Yang, C., Liang, W., Antonov, A., Cai, Y., Foss, F., Røskaft, E., 2012. Diversity of parasitic cuckoos and their hosts in China. Chinese Birds 3, 9–32. doi:10.5122/cbirds.2012.0004
